# Efficacy of Sodium-Glucose Cotransporter 2 Inhibitors in Patients With Concurrent Type 2 Diabetes Mellitus and Non-Alcoholic Steatohepatitis: A Review of the Evidence

**DOI:** 10.3389/fendo.2021.768850

**Published:** 2021-12-07

**Authors:** Koichi Yabiku

**Affiliations:** Division of Endocrinology, Diabetes and Metabolism, Hematology, Rheumatology (Second Department of Internal Medicine), Graduate School of Medicine, University of the Ryukyus, Okinawa, Japan

**Keywords:** non-alcoholic fatty liver disease, non-alcoholic steatohepatitis, sodium-glucose cotransporter 2 inhibitors, type 2 diabetes, review

## Abstract

Non-alcoholic fatty liver disease (NAFLD) is the most prevalent liver disease worldwide, and more than half of individuals diagnosed with type 2 diabetes concurrently present with NAFLD. There is a bidirectional pathological relationship between the two conditions, whereby NAFLD increases the risk of type 2 diabetes, and type 2 diabetes contributes to and accelerates the progression of NAFLD. Furthermore, over 30% of patients with NAFLD progress to non-alcoholic liver steatohepatitis (NASH), which then increases the risk of cirrhosis and hepatocellular carcinoma. Despite its high prevalence and the potential clinical implications, the underlying pathogenesis of NAFLD has yet to be fully elucidated, and there is no consensus regarding standard diagnosis and treatment for either NALFD or NASH. As patients with both NASH and type 2 diabetes have impaired hepatic function owing to chronic inflammation and the resulting structural changes caused by hepatic fat accumulation, they face reduced options for antidiabetic treatment. SGLT-2 inhibitors inhibit glucose reabsorption in the proximal tubule, with increased excretion of glucose in urine and decreased glucose levels in plasma, and their glycemia-lowering effect is insulin-independent. Several other beneficial effects have been reported for SGLT-2 inhibitors, including reduced risks of cardiovascular and renal diseases, improved blood pressure control, body weight reduction, and reductions in liver fat content. Experimental studies in mouse models have suggested that SGLT-2 inhibitors may have beneficial modulatory effects on NAFLD/NASH. Several trials in patients with type 2 diabetes have also suggested that these drugs may be useful in treating both type 2 diabetes and NAFLD or NASH. However, further research is needed to identify the mechanisms by which SGLT-2 inhibitors affect fatty liver and steatohepatitis. In this state-of-the-art review, we explore the literature on the efficacy of SGLT-2 inhibitors in patients with type 2 diabetes and NASH, and present arguments for and against the use of SGLT-2 inhibitors in this patient population.

## Introduction

Worldwide, the increasing prevalence of type 2 diabetes is occurring alongside a corresponding increase in the prevalence of non-alcoholic fatty liver disease (NAFLD). The co-existence of these two conditions is well known; the association is driven by a bidirectional pathological relationship whereby NAFLD increases the risk of type 2 diabetes, and type 2 diabetes contributes to, and accelerates, the progression of NAFLD (1). NAFLD is the most prevalent liver disease worldwide, affecting 25% to 30% of the global population (2). Globally, approximately 55% of patients with type 2 diabetes present with NAFLD, while in Japan, up to 68% of patients with type 2 diabetes concurrently present with NAFLD (3). NAFLD has several phases of progression, with approximately 20% to 30% of those who develop NAFLD progressing to non-alcoholic liver steatohepatitis (NASH) (4). Patients with NASH and concomitant type 2 diabetes are at an increased risk of progression to cirrhosis and hepatocellular carcinoma (5, 6).

NAFLD is commonly characterized by the presence of hepatic steatosis (with ≥5% of hepatocytes affected) without any sign of hepatocyte ballooning and in the absence of any secondary causes. Meanwhile, NASH is a more aggressive disorder that equally exhibits hepatic steatosis, but additionally—and crucially—includes hepatocyte ballooning (i.e., hepatocellular injury) and lobular necroinflammation with or without pericellular and perisinusoidal fibrosis (7, 8).

While patients with NAFLD may have no specific symptoms, some individuals may present with fatigue and right upper quadrant abdominal pain. Patients with NAFLD are also more likely to be obese and hypertensive and to show mild or moderate hepatomegaly. Common laboratory findings in NAFLD patients are hyperlipidemia, hyperglycemia, decreased insulin sensitivity, and mild or moderate elevations of liver enzymes, specifically, alanine and aspartate aminotransferases (ALT and AST) (9). The clinical presentation of NASH is similar to that of NAFLD (8); however, inflammation and hepatic injury must also be present for a diagnosis of NASH.

Currently, the gold-standard histological technique for detecting NAFLD and NASH is liver biopsy. This technique is highly invasive, prone to sampling errors, and may cause complications. Ultrasonography is the primary diagnostic imaging technique for NAFLD (1).

Although the pathogenesis of NAFLD has yet to be fully elucidated, several theories have been proposed. Most widely accepted is the two-hit theory (10), featuring hepatic lipid accumulation as the first hit and development of liver inflammation and fibrogenesis as the second. However, many investigators deem this model too simplistic, as several factors have been implicated in the development of NAFLD and NASH (11, 12). In particular, insulin resistance, imbalance of the lipid metabolism with adipose tissue dysfunction, hyperinsulinemia, lipo- and glucotoxicity, oxidative stress, and inflammation have been recognized as critical events in NAFLD development (6). In patients with insulin resistance, the effect of insulin is suppressed, leading to constant glucose output from the liver. Peripheral insulin resistance mainly occurs in adipose tissue and muscles, whereby glucose uptake and utilization mediated by insulin are disrupted, and there is an increased decomposition of adipose tissue. In skeletal muscle, there is a lack of glucose uptake and glycogen synthesis that leads to postprandial hyperglycemia. Consequently, the liver glucose uptake increases, triggering carbohydrate response element-binding protein (ChREBP) activation, a central metabolic coordinator, which stimulates intracellular glycolysis, contributing to ectopic *de novo* lipogenesis. In adipose tissue, insulin resistance interferes with the suppression of lipolysis. As a result of unrestrained lipolysis, triglycerides are hydrolyzed, releasing vast amounts of free fatty acids (FFAs) and glycerol. These circulating FFAs accumulate in the liver and are the primary substrates for hepatic triglyceride synthesis, eventually leading to intrahepatic triglyceride accumulation or steatosis. In the liver, selective hepatic insulin resistance increases gluconeogenesis and lipogenesis (13). Excess non-esterified FFAs released into the bloodstream lead to excessive uptake by tissues including the liver, heart, pancreas, muscle, and endothelial cells, causing significant organ dysfunction, i.e., lipotoxicity, which further promotes peripheral insulin resistance, hepatic gluconeogenesis, hyperglycemia, and pancreatic beta-cell dysfunction (14). Diacylglycerols, ceramides, and long-chain fatty acyl-coenzyme A (CoA) are toxic lipids that activate several inflammatory pathways. They can also cause direct damage resulting in hepatocellular injury and liver fibrosis (9) and indirectly increase oxidative stress, thereby triggering further inflammation and apoptosis (14).

Lipotoxicity is closely intertwined with glucotoxicity or chronically elevated plasmatic glucose concentrations causing cellular dysfunction and insulin resistance, and progressive metabolic deterioration is also associated with increased *de novo* lipogenesis. Both are major contributors to insulin resistance, beta-cell dysfunction, impaired insulin secretion, and ectopic fat deposits. Two pathways for *de novo* lipogenesis that result from chronically elevated glucose have been described. One is a direct pathway of increased tricarboxylic acid cycle activity and CoA synthesis, a substrate for *de novo* lipogenesis and gluconeogenesis (15). The other is an indirect pathway that involves increasing ChREBP expression in the liver and extrahepatic tissues, including the pancreas, kidney, and skeletal muscle. Additionally, liver X receptor α (LXRα), a ligand-activated transcription factor, is increasingly expressed, leading to elevated transcription of ATP-citrate lyase, fatty acid synthase and *SCD-1* (16). The active complex of LXRα and enhancer-binding protein β binds to the sterol regulator element-binding protein (SREBP)-1c at sites required for insulin induction (17).

The SREBP family plays a key role in lipid homeostasis, mainly that of cholesterol and triglycerides, as well as other important roles in the regulation of physiological functions of other organs (18). SREBP-1c, specifically, is expressed mainly in the brain, liver, white adipose tissue, skeletal muscle and adrenal glands of humans, and it is known to mediate, at least in part, insulin’s effects on hepatic lipogenesis (18, 19). Insulin increases the expression of SREBP-1c, it promotes the mobilization of the SREBP-cleavage activating protein (SCAP)/SREBP-1c complex to the Golgi apparatus, SREBP-1c cleavage, and the successive processes leading to hepatic lipogenesis and hepatic steatosis (20).

Oxidative stress, along with mitochondrial dysfunction, increased expression of pro-inflammatory cytokines and adipokines, and subsequent lipid peroxidation are relevant factors not only in the development of NAFLD (21) but also the progression to NASH (22). Adiponectin is said to have a protective effect in NAFLD by potentiating insulin’s ability to decrease glucose production and output, as well as down-regulating SREBP-1c and suppressing lipogenesis. Lipcalin-2 (LCN-2) and omentin-1, both newly discovered adipokines, seem to have a protective effect in NAFLD (23); however, LCN-2 may also contribute to insulin resistance. Leptin, conversely, is potently mitogenic on hepatic cells, inhibits cell apoptosis, and promotes processes involved in liver fibrosis (24). Other adipokines, such as resistin, seem to be involved in insulin resistance, while tumor necrosis factor alfa (TNF-α) and IL-6, which may be present at early and late stages of NAFLD, increase expression of SREBP-1c in the liver and further promote insulin resistance (21).

Recent studies have also shown that NAFLD may potentially occur in the absence of insulin resistance. Some individuals with single nucleotide polymorphisms within the patatin-like phospholipase 3 (*PNPLA3*) gene develop hepatic steatosis without first developing insulin resistance (22, 25). Clinical analyses have suggested that transcription factor 7–like 2 (*TCF7L2*) polymorphism modulates beta-cell function, lipid metabolism, glucose homeostasis, postprandial lipoprotein and adipokine responses, and hepatocyte apoptosis with the ingestion of fats. This polymorphism is associated with a predisposition to developing diabetes and liver injury leading to NAFLD and NASH among patients with familial dyslipidemia (26).

### Patient Population

Patients with NASH have impaired hepatic function owing to chronic inflammation and resulting structural changes caused by hepatic fat accumulation. Consequently, because of resultant drug hepatotoxicity, patients with type 2 diabetes and NASH face reduced options for antidiabetic treatment (27). For this reason, it has been suggested that concurrent type 2 diabetes and NAFLD require customized pharmacological management (28) to prevent the exacerbation or worsening of hepatic dysfunction.

**Figure 1:**
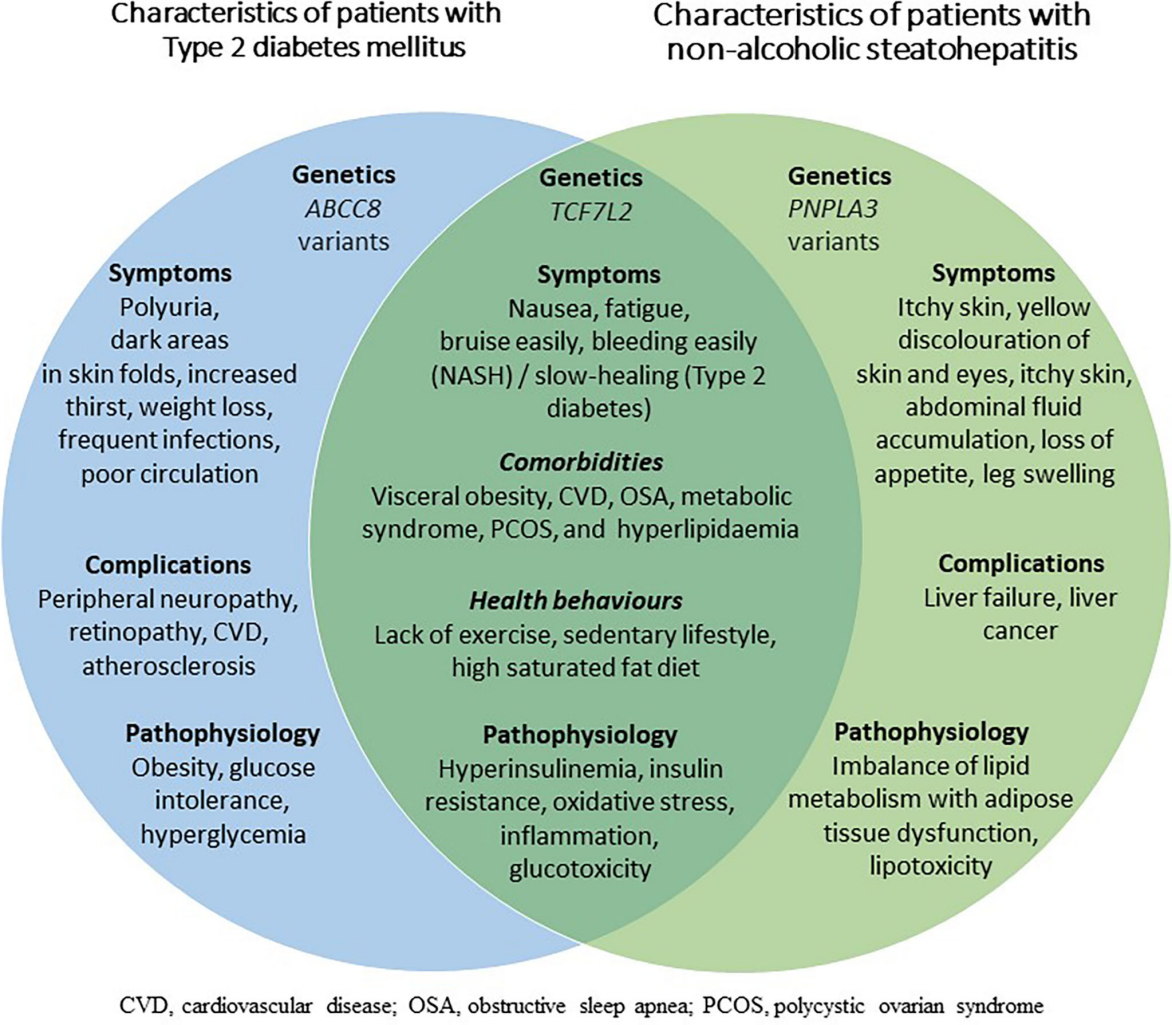
A Venn diagram of the symptoms, pathological features, and genetic features of type 2 diabetes and non-alcoholic steatohepatitis.

The published literature reveals that although the two disorders feature overlapping characteristics, patients with type 2 diabetes and NAFLD or NASH exhibit different pathophysiology than patients with type 2 diabetes only (illustrated in **Figure 1**). There are also important differences between patient populations with and without diabetes who develop NAFLD and progress to NASH. For instance, NASH in non-diabetic patients is associated with metabolic syndrome, with an increased risk of NASH in patients exhibiting more features of metabolic syndrome (29). Furthermore, patients with NAFLD and metabolic syndrome are significantly more likely to have marked insulin resistance and severe steatosis and portal inflammation as indicated by liver biopsies (30).

### SGLT-2 Function, Benefits of Its Inhibition in This Patient Population, and Possible Benefits in Combination With Other Antidiabetic Drugs

Sodium-glucose cotransporters (SGLTs) are responsible for glucose absorption from the small intestine as well as glucose reabsorption from the proximal tubule of the kidney (31, 32). As SGLTs actively transport glucose and sodium across membranes, glucose transport across the apical membrane and against the concentration gradient is driven by energy produced by the cotransport of sodium. While there are several SGLTs, SGLT-2 is responsible for more than 90% of the reabsorption of filtered glucose (33).

SGLT-2 inhibitors inhibit the reabsorption of glucose in the proximal tubule, resulting in increased glucose excretion in urine and decreased glucose levels in plasma. The resultant glycemia-lowering effect is insulin-independent (8). Aside from reducing hyperglycemia and increasing insulin sensitivity, several other beneficial clinical effects have been reported for SGLT-2 inhibitors, including reduced risks of cardiovascular and renal diseases, improved blood pressure control, body weight reduction, and reductions in liver fat content (34–37). In addition, experimental studies in mouse models suggest that SGLT-2 inhibition may have beneficial modulatory effects on NAFLD/NASH (38, 39). Of note, in an obese mouse model that exhibited characteristics of NAFLD, remogliflozin effectively improved ALT and AST levels and reduced hepatic triglyceride content (39). More recently, we investigated the effects of the SGLT-2 inhibitor dapagliflozin on glucose metabolism in a mouse model of NASH with concurrent type 2 diabetes. We demonstrated that dapagliflozin improved glycemic control and resolved ascites in the NASH mice compared with the untreated mice (40). Although further research is needed to identify the mechanisms by which SGLT-2 inhibitors affect fatty liver and steatohepatitis, several trials in patients with type 2 diabetes have suggested that these drugs may be useful in treating both type 2 diabetes and NAFLD or NASH (41–46).

Several review articles on the role of antidiabetic agents in the management of NASH have proposed hypothetical models of how the underlying mechanism of action of these drugs might improve NASH parameters (14, 47, 48). A very recent meta-analysis including a total of 1950 patients with type 2 diabetes with and without NAFLD concluded that SGLT-2 inhibitor treatment improved liver structure and function (49). In general, the proposed mechanism explaining the action of SGLT-2 inhibitors in NAFLD/NASH is based on the reduction of plasma glucose due to glucosuria, and reversal of glucotoxicity, as well as reductions in circulating insulin and body weight. These collectively result in decreased peripheral and hepatic insulin resistance, and lead, in turn, to a reduction of *de novo* lipogenesis in the liver. Plasma glucagon levels are directly increased by SGLT-2 inhibitors, which stimulates beta-oxidation and ketone production, thereby shifting to fatty acid metabolism and ketosis. Although gluconeogenesis may increase, this is counterbalanced by glucosuria. Together, these events hypothetically lead to a decrease in the lipid content in the liver (48).

While SGLT-2 inhibitors reduce the risk of cardiovascular diseases, other antidiabetic medications such as glucagon-like-1 (GLP-1) receptor agonists, and the thiazolidinediones such as pioglitazone, have also been shown to exert cardioprotective effects, albeit *via* very different mechanisms from SGLT-2 inhibitors. The cardioprotective effect of SGLT-2 inhibitors is attributed to its hemodynamic effects, which have an early onset: reduced blood pressure, decreased intravascular volume, and reduced aortic stiffness. The cardioprotective effect of GLP-1 receptor agonists results from antiatherogenic/anti-inflammatory mechanisms, which have a slow progressive onset, such as reduced plasmatic levels of inflammatory and oxidative stress markers, including IL-6, ICAM-1, nitrotyrosine, 8-iso prostaglandin F2α, MCP-1, and TNFα (50, 51). Although GLP-1 receptor agonists and SGLT-2 inhibitors act *via* different mechanisms of action, they share common effects such as weight loss (via appetite inhibition and increased excretion of calories in the urine, respectively), amplification of beta-cell function (mediated by GLP-1 receptor and reversal of glucotoxicity, respectively), increased insulin sensitivity owing to weight loss and reversal of glucotoxicity, blood pressure reduction (natriuresis and reduction of intravascular volume), anti-inflammatory effects (mainly GLP-1 receptor agonists), natriuresis, protection against diabetic renal complications, and modest effects on lipid profile. Thus, it is expected that this combination could result in additive benefits, such as enhanced peripheral insulin sensitivity, greater reduction in systolic/diastolic blood pressure, and possibly prevention of diabetic glomerulosclerosis compared with monotherapy. Further, as both GLP-1 receptor agonists and SGLT-2 inhibitors reduce visceral fat, and GLP-1 receptor agonists reduce fat deposits in the liver in patients with NAFLD/NASH (52, 53), this drug combination may be instrumental in the treatment of NAFLD/NASH.

Another class of antidiabetic drugs comprises the thiazolidinediones, which are peroxisome proliferator-activated receptor gamma (PPARγ) agonists. While three isoforms of PPAR exist (alpha [α], beta/delta [β/δ] and γ), PPARγ is most highly expressed in adipose tissue, with even greater expression observed in the liver of patients with NAFLD. The activation of PPARγ results in the synthesis of several adipokines, including adiponectin, which promote FA oxidation, storage of fat in adipose tissue with consequent reduction of lipolysis, and less accumulation of fat in the liver. Although the preclinical evidence obtained from mouse models was promising, clinical evaluation of thiazolidinediones in patients with NASH or NAFLD produced variable outcomes or did not reach statistical significance. However, several meta-analyses of randomized controlled trials have concluded that pioglitazone, although less potent than rosiglitazone, is superior in suppressing hepatic fibrosis and inflammation along with the reversal of steatosis (54). More recent re-evaluations of previously published evidence have suggested that in addition to its glucose-lowering effects, pioglitazone also has cardioprotective effects (55, 56); however, the mechanism underlying these effects remains unclear (56). Pioglitazone exerts a robust protective effect against atherosclerosis-driven cardiac and cerebrovascular events; in preclinical and clinical studies, pioglitazone reduced left ventricular systolic and diastolic dimensions and improved left systolic and diastolic functions. Pioglitazone also directly benefits cardiomyocyte electrophysiology, energetic metabolism, ischemia-reperfusion injury, cardiac remodeling, neurohormonal activation, and pulmonary circulation. However, adverse events related to increases in body weight, peripheral circulation, congestive heart failure, and bone mineral density, among others, must be considered when prescribing this drug (57). A clinical study comparing SGLT-2 inhibitor tofogliflozin vs pioglitazone showed that hepatic steatosis significantly decreased in both groups after 24 weeks of treatment, but to a greater extent with pioglitazone than with tofogliflozin. While tofogliflozin reduced body weight, pioglitazone increased body weight from baseline. However, it is possible that combining both drugs could potentially result in an additive effect on hepatic steatosis and could balance out the effects of pioglitazone on body weight gain (58). A recent preclinical study using a mouse model of type 2 diabetes showed that a combination of the SGLT-2 inhibitor ipragliflozin plus pioglitazone significantly improved multiple NASH parameters, including hyperglycemia, insulin resistance, hyperlipidemia and liver injury (hepatic steatosis and fibrosis). Moreover, these parameters were additively improved with combination treatment versus ipragliflozin monotherapy (59).

### Objectives

This state-of-the-art review explores the current literature reporting the efficacy of SGLT-2 inhibitors in patients with type 2 diabetes and NASH and presents arguments for and against the use of SGLT-2 inhibitors in this patient population, where it plays a regulatory role in adipocyte differentiation, adipogenesis, and lipid metabolism.

## Disease Management

Ultimately, the goal of NASH treatment is to arrest and revert steatohepatitis in order to stop the progression of hepatocellular injury and fibrosis. Additionally, and ideally, the risk of cardiovascular events should also be reduced with the selected treatment, regardless of the presence or absence of diabetes. Although there is an increasing need for effective treatments for NAFLD and NASH, until very recently, no specific on-label treatment had been approved or recommended (60). Furthermore, treating patients with both type 2 diabetes and NAFLD or NASH is complex and challenging because of the various comorbidities that frequently exist in these patients (61), and data from clinical trials of antidiabetic treatment typically lack high-quality and clinically relevant endpoints related to liver histology. Of note, saroglitazar, a dual PPARα/γ agonist, was approved in India in March 2020 for the treatment of NASH. However, at the time of writing this review, there are currently no drugs approved by the FDA or European Medicine Agency for this disease. Nevertheless, several drugs being developed for the treatment of NASH are currently in advanced phase 3 clinical trials (e.g., obeticholic acid, cenicriviroc, aramchol, resmetirom, dapagliflozin, and semaglutide). Moreover, others in the pipeline (e.g., elapectin, MSDC-0602K, lanifibranor, efruxifermin, and tesamorelin) are expected to commence late-phase trials in the near future (62).

The primary recommendations for managing patients with type 2 diabetes and concurrent NAFLD or NASH who are overweight or obese include lifestyle modifications (e.g., increased exercise (63) and dietary adjustments) to achieve sustained weight loss. Weight loss of 5% or more has been shown to reduce steatosis and improve liver enzyme levels, glycemic control, and insulin sensitivity. Moreover, weight loss of 7% or more has been associated with NASH improvements, including reduced fibrosis (1, 64). Other recommendations tend to focus on managing the presence of comorbidities. Surgical treatments such as bariatric surgery and liver transplants are high-cost treatment alternatives, with limited evidence regarding efficacy and safety. Furthermore, these treatments are only accessible to a limited number of patients (65).

As noted in Section 1, several off-label treatments have been explored as potentially suitable therapies for patients with NAFLD and NASH. For example, the efficacy of insulin-sensitizing medications, such as thiazolidinediones and metformin, has been assessed in small randomized controlled trials (66). Of these, pioglitazone is the most studied drug in patients with NAFLD and remains the only recommended medication for NAFLD or NASH (although it should be noted that this is off-label usage) (67). We previously compared the effects of six-month treatments with pioglitazone, metformin, sitagliptin, or a non-oral antidiabetic drug (control) combined with guidance on diet and exercise in a cohort of men with type 2 diabetes and NAFLD. Pioglitazone was found to yield the most marked improvements in liver fat levels (68). Importantly, pioglitazone must be prescribed with caution as thiazolidinediones tend to exhibit a range of adverse effects such as weight gain, peripheral edema, cardiac failure, bladder cancer, and bone fragility (69). Other antidiabetic agents which have shown promising preliminary results include the GLP-1 receptor agonists (liraglutide, exenatide, dulaglutide, and semaglutide). A recently published meta-analysis of pooled data from 11 randomized controlled trials of GLP-1 receptor agonists in patients with NAFLD or NASH indicated significant improvements in liver enzyme levels and liver fat content following treatment (70). In addition, several other therapies have been trialed, including antioxidants (e.g., vitamin E), lipid-lowering drugs (e.g., statins, ezetimibe, and peroxisome proliferator-activated receptor-α agonists), pentoxifylline, ursodeoxycholic acid, angiotensin receptor blockers, and n-3 polyunsaturated fatty acids. However, further evidence on the efficacy and safety of these treatments in randomized controlled trials is required before they can be granted regulatory approval for the treatment of NAFLD or NASH (71).

SGLT-2 inhibitors comprise a new family of antidiabetic drugs, of which ipragliflozin achieved the first global approval for clinical use in Japan in 2014 to treat type 2 diabetes (72). There are now seven SGLT-2 inhibitors that have been approved and are currently used in Japan (i.e., ipragliflozin, dapagliflozin, tofogliflozin, luseogliflozin, canagliflozin, ertugliflozin, and empagliflozin). In contrast, only four SGLT-2 inhibitors (dapagliflozin, canagliflozin, empagliflozin, and ertugliflozin) have been approved by the Food and Drug Administration (FDA) in the United States. Aside from their hypoglycemic effects, these antidiabetic drugs are known to have cardiovascular, renal, and hepatic extraglycemic benefits (73). Several pilot studies have been conducted in Japan to determine the efficacy and safety of SGLT-2 inhibitors for patients with type 2 diabetes and NAFLD or NASH (74–77). These studies showed that patients with NAFLD achieved significant reductions in ALT levels, body weight, and fatty liver index. A phase 3 trial of dapagliflozin efficacy and action in NASH (DEAN; ClinicalTrials.gov Identifier NCT03723252) is currently ongoing and aims to assess changes in liver histology as well as various metabolic parameters in patients with NASH. Recently, a meta-analysis of randomized trials evaluated 1950 patients with type 2 diabetes with and without NAFLD and concluded that SGLT-2 inhibitor treatment improved liver structure and function (49). **Table 1** lists the typical outcomes measured in the studies conducted to date; these include changes in serum aminotransferase levels, NASH histology scores, and liver fat levels. Additional details including the intervention evaluated, the characteristics of the patient population, and the study duration can be found in **Table 2**. These outcome measures are generally indicative of the condition of the liver, although some are more sensitive than others. For instance, levels of transaminases should be interpreted with care as some patients with NAFLD or NASH can still present with normal ALT levels. Furthermore, improvements in liver histology after SGLT-2 inhibitor treatment may not be reflected in decreased liver enzymes (34). It is also very important to consider how SGLT2 inhibitors affect the expression of lipid metabolism genes, such as *ACC*, *SREBP-1c*, *CPT1a*, and *PPARα*, in the liver. Recently, hepatic mitochondrial dysfunction has attracted attention as a common factor underlying both diabetes mellitus and NAFLD/NASH (81); therefore, it is critical to elucidate how SGLT2 inhibitors might impact mitochondrial function. The typical monitoring of patients on SGLT-2 inhibitors to treat this dual condition includes metabolic blood panels (liver enzymes, HbA1c, and fasting plasma glucose) and radiological imaging, including ultrasound, computed tomography, magnetic resonance imaging (MRI), MR spectroscopy, and vibration-controlled transient elastography.

**Table 1:** Outcome measures commonly recorded in studies of the efficacy of SGLT-2 inhibitors in patients with concurrent type 2 diabetes mellitus and non-alcoholic steatohepatitis.

Index	Measured as change from baseline	Measured as change from placebo
Fasting plasma glucose	Eriksson et al., (41)	Ohki et al., (78)
Serum alanine aminotransferase	Eriksson et al., (41)	Ito et al., (42) Shibuya et al., (79)
Serum aspartate transaminase	Eriksson et al., (41)	Seko et al., (74) Gautam et al., (80)
Serum gamma-glutamyl transferase	Eriksson et al., (41)	Kuchay et al., (34) Seko et al., (74)
Glycosylated hemoglobin	Eriksson et al., (41)	Ohki et al., (78) Shibuya et al., (79) Kuchay et al., (34)
Body mass index	Gautam et al., (80)	Ito et al., (42) Shibuya et al., (79)

SGLT-2, sodium-glucose transport protein 2.

**Table 2:** Intervention, interventional group characteristics, and duration of studies exploring the efficacy of SGLT-2 inhibitors in patients with concurrent type 2 diabetes mellitus and non-alcoholic fatty liver disease.

Study	Intervention	Interventional group characteristics	Duration
Eriksson et al., (41)	Dapagliflozin	Type 2 diabetes, aged 40–75 years, BMI 25–40 kg/m^2^	12 weeks
Gautam et al., (80)	Canagliflozin	NAFLD, type 2 diabetes, abnormal LFT	6 months
Ohki et al., (78)	Ipragliflozin	NAFLD and type 2 diabetes	Median 320 days
Ito et al., (42)	Ipragliflozin	NAFLD and type 2 diabetes	24 weeks
Shibuya et al., (79)	Luseogliflozin	NAFLD and type 2 diabetes, aged 47–62 years, BMI 26.2–28.7 kg/m^2^	Median 5 weeks
Seko et al., (74)	Ipragliflozin, Canagliflozin	NAFLD and type 2 diabetes	12 weeks
Kuchay et al., (34)	Empagliflozin	NAFLD and type 2 diabetes, aged >20 years	20 weeks

BMI, body mass index; LFT, liver function test; NAFLD, non-alcoholic fatty liver disease; SGLT-2, sodium-glucose transport protein 2.

## Comparative Efficacy of SGLT-2 Inhibitors in Patients With Type 2 Diabetes and NASH

Although formal head-to-head studies are lacking, we have used data from the published literature to conduct a comparative analysis of the efficacies of dapagliflozin, canagliflozin, empagliflozin, ertugliflozin, ipragliflozin, luseogliflozin, and remogliflozin (**Table 3**). When using MRI to observe change in liver fat content, dapagliflozin combined with omega-3 carboxylic acids improved NAFLD in a randomized, controlled, double-blind trial of patients with type 2 diabetes (41). Furthermore, in patients with concurrent type 2 diabetes and NAFLD who were already receiving standard of care for type 2 diabetes, add-on empagliflozin therapy significantly reduced liver fat compared with standard treatment alone (34).

**Table 3:** Summary of Japanese clinical and preclinical studies of SGLT-2 inhibitors for type 2 diabetes with or without NAFLD/NASH.

First author	Year	Study type	Subjects (n) Subjects (n)	Duration (weeks)	SGLT-2 intervention	Study arms	Findings	Conclusions
***Clinical studies***								
**Akuta**	2017	Observational (prospective)	5	24	Canagliflozin	Canagliflozin 100 mg/day	Treatment improved histopathologic features in all five patients with type 2 diabetes and NASH.	Canagliflozin improves histopathologic markers of liver function in this patient population.
**Seko**	2017	Observational (retrospective)	45	24	Ipragliflozin, canagliflozin	DPP-4 inhibitor versus SGLT-2 inhibitor (canagliflozin 100 mg/day or ipragliflozin 50 mg/day)	SGLT-2 inhibitors reduced body weight and HbA1c, body mass index, and plasma glucose.	SGLT-2 inhibitors are promising treatments in this patient population.
**Seko**	2018	Observational (prospective)	10	12	Ipragliflozin, canagliflozin	Canagliflozin 100 mg/day	Several hepatic function/fibrosis markers improved, and serum alanine aminotransferase levels decreased.	Canagliflozin is effective and well-tolerated in this patient population.
**Komiya**	2016	Observational (prospective)	55	24	Ipragliflozin	Ipragliflozin 50 mg/day; results stratified by hepatic steatosis status	Ipragliflozin improved liver function in patients with type 2 diabetes regardless of whether the patient lost weight or not.	The preclinical results in mice in this study suggest that ipragliflozin can attenuate insulin resistance.
**Ito**	2017	RCT (open-label)	66	24	Ipragliflozin	Ipragliflozin 50 mg/day versus pioglitazone 15–30 mg/day	Both drugs exhibited equally beneficial effects on NAFLD and glycemic control in patients with type 2 diabetes and NAFLD, but ipragliflozin also reduced body weight and abdominal fat.	SGLT-2 inhibitors may be as effective as thiazolidinediones in this patient population, with added benefits.
**Ohki**	2016	Observational (retrospective)	130	Median 320 days	Ipragliflozin	Ipragliflozin and a DPP-4 inhibitor versus ipragliflozin and a GLP-1 analog	Body weight, HbA1c, and ALT levels decreased significantly.	Adding ipragliflozin improves glycemic control, leads to weight loss, and normalizes indices of liver function.
**Takase**	2017	Observational	21	16	Ipragliflozin	Ipragliflozin 15 mg/day	Ipragliflozin decreased body weight, HbA1c, fatty liver indices, adipose tissue, and fat mass; better glucose tolerance was also achieved.	Ipragliflozin reduces markers of fatty liver.
**Takeda**	2017	Case report	1	16	Ipragliflozin	N/A	Serum ALT and ferritin levels decreased to normal after 4 months of treatment; liver fat deposits, type IV collagen, and hyaluronic acid also decreased after treatment.	Ipragliflozin may be effective in treating NASH.
**Seino**	2014	RCT (open-label)	158	24	Luseogliflozin	Luseogliflozin 2.5 mg/day versus placebo	Luseogliflozin reduced HbA1c, body weight, and abdominal circumference.	Luseogliflozin monotherapy is beneficial and well-tolerated in Japanese patients with type 2 diabetes.
**Shibuya**	2018	RCT (pilot trial)	32	24	Luseogliflozin	Luseogliflozin 2.5 mg/day versus metformin 1500 mg/day	Changes from baseline were greater in the luseogliflozin group.	Luseogliflozin was more effective than metformin at reducing liver fat deposition.
**Sumida**	2019	Single-arm (prospective)	40	24	Luseogliflozin	Luseogliflozin 2.5 mg/day	Luseogliflozin treatment decreased liver fat content and HbA1c.	Luseogliflozin is a promising treatment in this patient population.
***Preclinical studies***								
**Yabiku**	2020		560 C57BL/6J mice	2	Dapagliflozin	Dapagliflozin (1 mg/kg/day) or furosemide (30 mg/kg/day) versus vehicle	Dapagliflozin improved glucose tolerance.	Dapagliflozin does not seriously affect hemodynamics in mice and may be suitable in a patient population with type 2 diabetes and NASH.
**Jojima**	2016		28 C57BL/6J mice	3	Empagliflozin	Linagliptin (10 mg/kg); empagliflozin (10 mg/kg); and linagliptin (10 mg/kg) and empagliflozin (10 mg/kg); versus vehicle	Empagliflozin or empagliflozin and linagliptin lowered fatty liver disease activity scores and reduced hepatic inflammatory markers more than linagliptin alone; the combined treatment reduced collagen deposition.	A combined treatment ameliorates NASH better than the administration of either empagliflozin or linagliptin.
**Tahara**	2013		12 C57BL/6J mice	4	Ipragliflozin	Ipragliflozin (0.3 mg/kg/day, 1 mg/kg/day, and 3 mg/kg/day) versus vehicle	Ipragliflozin reduced markers of insulin resistance, oxidative stress, and inflammation.	Ipragliflozin may be useful in treating metabolic syndrome.
**Yokono**	2014		16 C57BL/6J mice	4	Ipragliflozin	Ipragliflozin (10 mg/kg/day) versus vehicle	Treatment reduced body weight and fat mass even though food intake increased.	Ipragliflozin may enhance lipolysis and fatty acid oxidation and promote energy utilization from fat rather than glucose.
**Qiang**	2015		C57BL/6J mice (number not stated)	8	Luseogliflozin	High-fat diet versus a high-fat diet with luseogliflozin (0.1%)	Changes in glucose levels, liver weight, and hepatic lipid accumulation were smaller in the group of diabetic mice that received luseogliflozin.	Luseogliflozin may be appropriate in patients with diabetes to prevent or delay the development of NASH.
**Nakano**	2015		20 C57BL/6J mice	15	Remogliflozin	Normal diet or a high-fat diet versus a high-fat diet with remogliflozin	Treatment reduced liver injury and oxidative stress markers and levels of hepatic triglycerides in obese mice.	Remogliflozin may have insulin-sensitizing and antioxidant properties.
***Meta-analyses***								
**Sumida**	2020	Review	N/A	N/A	Ipragliflozin, empagliflozin, remogliflozin, luseogliflozin, dapagliflozin, ertugliflozin	N/A	A brief summary of the findings of 15 studies, without synthesizing the findings.	SGLT-2 inhibitors are cardioprotective and renoprotective in this patient population.

ALT, alanine aminotransferase; DPP-4, dipeptidyl peptidase-4; NAFLD, non-alcoholic fatty liver disease; NASH, non-alcoholic steatohepatitis; RCT, randomized controlled trial; SGLT-2, sodium-glucose transport protein 2; N/A, not applicable.

In an observational study in India, it was shown that canagliflozin 100 mg resulted in liver function improvements and reductions in body weight in patients with type 2 diabetes and NAFLD (80). Similarly, ipragliflozin has also been shown to reduce body weight in addition to abdominal fat in a study of ipragliflozin versus pioglitazone, although both drugs showed equal benefits for NAFLD and glycemic control (42). However, a more recent single-arm prospective trial has reported that luseogliflozin 2.5 mg/day significantly reduced HbA1c, transaminases, and hepatic fat content after 24 weeks of treatment (27). This supports previous results observed during a randomized pilot study of luseogliflozin 2.5 mg/day, which resulted in significantly larger reductions in fat liver deposition compared with metformin 1500 mg/day (79). Finally, a study of NAFLD patients with type 2 diabetes who did not respond to incretin-based therapy showed that SGLT-2 inhibitor treatment resulted in good glycemic control, reduced body weight, normalized ALT levels, and a reduced Fibrosis-4 index (78). This was followed by a single-arm, exploratory study of canagliflozin, where it was reported that serum ALT levels decreased, and several markers of hepatic function and fibrosis also improved (74).

One of the main advantages of SGLT-2 inhibitor monotherapy is the ability of a single drug to provide better glycemic control and reduce insulin resistance whilst also improving NAFLD features and reducing body weight. SGLT-2 inhibition occurs independently from β-cell function and secretion of insulin; thus, these agents can be useful for glycemic control for patients with longstanding diabetes with or without preserved renal function (82). Moreover, treatment with SGLT-2 inhibitors has been reported to be well-tolerated, with a low risk of hypoglycemia (on par with the risks posed by metformin and DPP-4 inhibitors) (83). Indeed, a fairly recent meta-analysis evaluated the benefits and harms of SGLT-2 inhibitors and concluded that the risk of adverse effects while receiving treatment with these agents was comparable to that associated with placebo treatment (84).

However, SGLT-2 inhibition monotherapy has some limitations. One important limitation is that these drugs appear to be more effective in younger individuals with a shorter duration of diabetes and higher baseline BMI, fasting glucose level, and HbA1c level (83, 85). Additionally, many of the relevant studies of SGLT-2 inhibitors have been conducted among Japanese patients, and as such, there is a clear need for studies in other populations. Previously, SGLT−2 inhibition was contraindicated for patients with impaired renal function (estimated glomerular filtration rate [eGFR] <45 mL/min/1.73 m^2^), and thus, this treatment was limited to patients with diabetes and preserved kidney function (83). However, given recent favorable effects in major clinical trials (e.g., CANVAS, CREDENCE, DAPA-CKD) (86–88), both canagliflozin and dapagliflozin have been approved by the FDA for use in patients with chronic kidney disease. Empagliflozin was also approved by the FDA for use in patients with heart failure (regardless of diabetes status) and chronic kidney disease (eGFR ≥20 to < 45 mL/min/1.73 m^2^), on the basis of findings from the EMPA-KIDNEY trial (82). Moreover, accumulating evidence supports a strong relationship between NAFLD and diabetic nephropathy.

The most frequently reported adverse effects of SGLT-2 inhibitors are mycotic genital infections, which are attributable to the presence of glucosuria and the resulting proliferation of pathogens. Urinary tract infections and volume depletion effects are reportedly rare, and these effects are also related to glucosuria, which can lead to osmotic diuresis (83). Concerns have been raised regarding the risk of euglycemic ketoacidosis, bone fracture, and foot and leg amputation (89–91). Specific caution is warranted when prescribing SGLT-2 inhibitors to frail elderly patients aged >75 years, particularly those with chronic kidney disease and those using loop diuretics (89), as the concomitant use of loop diuretics and SGLT-2 inhibitors can increase the risk of volume depletion (92).

Overall, however, the existing literature implies that the benefits of SGLT-2 inhibitors in patients with type 2 diabetes and NASH far outweigh the potential adverse effects. Combination therapies such as canagliflozin/metformin, dapagliflozin/metformin, empagliflozin/metformin, and empagliflozin/linagliptin, which may also be useful in this patient population, have been approved in several countries. The complementary therapeutic advantages include beneficial metabolic effects that result from the SGLT-2 inhibitor and improvements in insulin sensitivity provided by metformin. However, it is important to recognize that some patients may be more susceptible to adverse events associated with these combination therapies. It is also plausible that combining an SGLT-2 inhibitor with pioglitazone could be a useful approach with favorable effects on glucose homeostasis, insulin sensitivity, and bodyweight. Furthermore, this combination therapy could reduce adverse effects, as the diuretic effect of SGLT-2 inhibitors would counteract the fluid retention caused by the thiazolidinediones. Finally, it has also been proposed that SGLT-2 inhibitors could be combined with a GLP-1 receptor agonist (93) or a DPP-4 inhibitor (38), resulting in additive effects due to these drugs’ different mechanisms of action (48). Treatment for diabetes and NASH with a combination of pioglitazone and either a GLP-1 receptor agonist or SGLT-2 inhibitor may be a cost-effective strategy while lowering the increased cardiovascular risks in this patient population (94).

A recently conducted meta-analysis of eight studies (214 patients in total) of patients receiving SGLT-2 inhibitors for type 2 diabetes mellitus and NAFLD (43) concluded that despite the low-to-moderate quality of evidence from the available pilot and observational studies, SGLT-2 inhibitors appear to improve transaminase levels, fatty liver, and fibrosis, along with providing additional metabolic effects, with the most common adverse effects being genitourinary tract infections. Another recent meta-analysis of 29 randomized controlled trials (2,617 patients in total) focused on treating NAFLD with antihyperglycemic drugs in patients with or without diabetes (70). Of the 29 reviewed studies, seven involved the administration of SGLT-2 inhibitors, whereas the other studies featured treatment with metformin, glitazones, GLP-1 receptor agonists, and DPP-4 inhibitors. Although most randomized controlled trials had adequate liver histological endpoints, most of the efficacy data produced in patients with NASH were for pioglitazone. Both systematic reviews (43, 70) suggest that further research is needed, particularly in the form of large, prospective, phase 3, randomized controlled trials, with multi-racial/multi-ethnic populations and widely accepted histologic endpoints. Nevertheless, many of the preliminary studies conducted to date have reported encouraging results, and it is hoped that future studies of SGLT-2 inhibitors in patients with type 2 diabetes and NAFLD can provide robust evidence to confirm their therapeutic potential within this population.

## Knowledge Gaps and Research Needs

At present, the exact pathogenic mechanisms of NAFLD and its progression to NASH in the presence or absence of diabetes remain unclear. To design optimal treatment strategies, it is necessary to fully understand the underlying pathogenesis of this dual condition. Moreover, the mechanisms of action of SGLT-2 inhibitors also need to be elucidated. This could be achieved by studies targeting molecular pathways that potentially mediate the therapeutic effects of SGLT-2 inhibitors and by leveraging state-of-the-art approaches, such as transcriptomics, lipidomics, and metabolomics. Pharmacogenomics testing may be beneficial in distinguishing extensive from poor metabolizers of SGLT-2 inhibitors, enabling the individualization of dosing for optimal therapeutic responses. However, this will require the identification of the metabolic enzymes that biotransform this drug class.

The main issues emerging from the two systematic reviews undertaken to date (43, 70) were that existing clinical studies varied considerably in design, interventions, and follow-up periods. Furthermore, most studies were conducted in single racial groups or limited geographic areas. Further research is needed to validate the reported benefits of SGLT-2 inhibitors in treating NAFLD and NASH. Therefore, future study designs should be prospective and allow comparisons of the long-term efficacy and safety profiles of the seven available SGLT-2 inhibitors. Samples should be adequately powered to provide clinically and statistically meaningful results, racially diverse, and include elderly (≥65–74 years) and very elderly (≥75 years) patients. In addition, future studies should evaluate appropriate and validated histologic outcomes as well as surrogate markers and imaging indices.

## Conclusions

This review has highlighted the challenges faced in the treatment of patients with concurrent type 2 diabetes mellitus and NAFLD or NASH, including the impact of comorbidities, lack of efficacy, and long-term safety issues. An assessment of the published literature suggests that SGLT-2 inhibitor monotherapy offers a promising option for the treatment of concurrent type 2 diabetes mellitus and NAFLD or NASH. Preliminary data show that this treatment is effective and well-tolerated in the populations studied, which mainly comprised Japanese patients. However, the available studies had several limitations that need to be addressed to solidify these findings. Combination therapies with SGLT-2 inhibitors warrant further study but may provide a feasible alternative for patients who are unresponsive to monotherapy or treatment with other drug classes. Moreover, the pharmacotherapy of patients with concurrent type 2 diabetes mellitus and NAFLD or NASH should be tailored to individual needs and requires a multidisciplinary approach. Further non-invasive diagnostic and follow-up measures are needed to improve the clinical management of this dual condition and optimize drug therapy. Given the incompletely understood pathogeneses of NAFLD and NASH, it is difficult to identify specific therapeutic targets. This issue needs to be addressed to enable optimization of treatment and outcomes in this rapidly growing patient population.

## Author Contributions

The author confirms being the sole contributor of this work and has approved it for publication.

## Conflict of Interest

The author declares that the research was conducted in the absence of any commercial or financial relationships that could be construed as a potential conflict of interest.

## Publisher’s Note

All claims expressed in this article are solely those of the authors and do not necessarily represent those of their affiliated organizations, or those of the publisher, the editors and the reviewers. Any product that may be evaluated in this article, or claim that may be made by its manufacturer, is not guaranteed or endorsed by the publisher.
